# Complete genome sequence of *Lacticaseibacillus paracasei* A02

**DOI:** 10.1128/mra.00082-24

**Published:** 2024-05-02

**Authors:** Jiahao Li, Xiuyun Wu, Sheng Xing, Bo Ding

**Affiliations:** 1 The State Key Laboratory of Microbial Technology, Shandong University, Qingdao, Shandong, China; ^2^Shan Dong Institute for Food and Drug Control, Jinan, Shandong, China; 3 NMPA Key Laboratory for Research and Evaluation of Generic Drugs, Jinan, Shandong, China; University of Maryland School of Medicine, Baltimore, Maryland, USA

**Keywords:** genome sequence

## Abstract

*Lacticaseibacillus paracasei,* a facultative anaerobic gram-positive bacterium, is commonly found in the gut of humans and animals, as well as in dairy products and plant ferments. Here, we report the complete genome sequence of *L. paracasei* A02, whose total genome length is 3,038,472 bp, with a GC content of 46.41%.

## ANNOUNCEMENT

*Lacticaseibacillus paracasei* is a species of facultative anaerobic gram-positive bacteria and commonly found in the gut of humans and animals, dairy products, and plant ferments (1). The *L. paracasei* A02 was isolated from stool sample of healthy children stored in – 80°C refrigerator. We adhered to the principles of the Declaration of Helsinki. An amount of 0.1 g sample was added to 1 mL 0.9% sterile sodium chloride solution. Also, 0.1 mL of the mixture was inoculated in 10 mL of Li-Mupirocin-modified Man Rogosa Sharpe (MRS) medium (QingDao Hopebio-Technology Co., Ltd.) and cultured at 37°C for 72 h under anaerobic conditions. The enriched bacterial solution with sterile saline gradient dilution was coated on an AGAR plate containing MRS medium. A single colony purified by three rounds of scribing was selected for further identification. The 16S rRNA gene sequence was amplified using PCR with the primer pair 27F and 1492R. The 16S rRNA gene was obtained (with the GenBank accession number PP439829) by Sanger dideoxy sequencing. The strain A02 was identified as *L. paracasei*.

*L. paracasei* A02 was cultured in Li-Mupirocin-modified MRS medium at 37°C for 48 h under anaerobic conditions for DNA isolation using the QIAGEN Genomic-tip 20/G Kit (QIAGEN). Oxford Nanopore Technologies (ONT) and Illumina libraries were prepared from the same DNA preparation. For Illumina sequencing, DNA samples were sheared into 400–500 bp fragments using a Covaris M220 Focused Acoustic Shearer following manufacturer’s protocol. Then, the library was prepared from the sheared fragments using the TrueLib DNA Library Rapid Prep Kit and sequenced on an Illumina Novaseq 6000 platform. For ONT sequencing, DNA fragments were repaired, purified, and then attach sequencing adapters supplied in the SQK-LSK109 Kit to the DNA ends. Then, the library was sequenced on a PromethION48 sequencer with PromethION Flow Cell (R9 Version). Illumina sequencing generated 4,644,856× 2 raw pair reads, 1,390,758,874 bases, with 99.04% coverage base on reads mapping and raw Q30 of 95.23%. The raw reads generated from the paired-end library were subjected to quality filtering using fastp v0.23.0. ONT sequencing generated 186,597 reads, 792,889,124 bases, with 98.07% coverage base on reads mapping and the largest, average length and reads N50 value of 112,940, 4249.21, and 8,408 bp. ONT reads were extracted, base called and demultiplexed, and trimmed with the minimum *Q*-score cutoff of 7. Guppy v5.0.16 was used as the base caller (2). Unicycler v0.4.8 (3) was used for hybrid assembly of Illumina data after quality control and ONT data, and finally, Pilon v1.22 (4) was used to map Illumina short sequences to the assembled genome for correction. Genome annotation was performed using NCBI Prokaryotic Genome Annotation Pipeline (PGAP v5.3) (5). Default parameters were used for all software unless otherwise specified.

The complete genome sequence of *L. paracasei* A02 contained one chromosome (length, 3,020,466 bp; GC content, 46.45%) and two plasmids (plasmid A: length, 11,833 bp; GC content, 40.69%; plasmid B: length, 6,173 bp; GC content, 39.28%). The total genome length is 3,038,472 bp, containing 2,823 protein-coding genes, 59 tRNAs, 15 rRNAs, and the average GC content is 46.41%.

## Data Availability

The genome sequence of *L. paracasei* A02 is available in NCBI GenBank under genome accession numbers CP139216-CP139218. The raw sequence data are available under Sequence Read Archive (SRA) accession numbers SRR27324570 (Illumina) and SRR27324569 (ONT).
